# Psychosoziale Unterstützung während der COVID-19-Pandemie: interdisziplinäres Versorgungskonzept an einem Universitätsklinikum

**DOI:** 10.1007/s00115-020-01014-8

**Published:** 2020-10-06

**Authors:** Matthias A. Reinhard, Gerrit Burkhardt, Fabienne Grosse-Wentrup, Daniela Eser-Valerie, Friederike H. A. Mumm, Bernhard Barnikol-Oettler, Claudia Bausewein, Michael von Bergwelt-Baildon, Matthias Klein, Peter Falkai, Karl-Walter Jauch, Kristina Adorjan, Frank Padberg, Eva Hoch

**Affiliations:** 1 Klinik für Psychiatrie und Psychotherapie, LMU Klinikum, Nußbaumstraße 7, 80336 München, Deutschland; 2 Medizinische Klinik und Poliklinik III, LMU Klinikum, München, Deutschland; 3 Seelsorge, LMU Klinikum, München, Deutschland; 4 Klinik und Poliklinik für Palliativmedizin, LMU Klinikum, München, Deutschland; 5 Zentrale Notaufnahme, LMU Klinikum, München, Deutschland; 6 Neurologische Klinik und Poliklinik, LMU Klinikum, München, Deutschland; 7 Klinik für Allgemeine, Viszeral, Transplantations- Gefäß- und Thoraxchirurgie, LMU Klinikum, München, Deutschland

**Keywords:** COVID-19-Pandemie, Psychosoziale Versorgung, Psychosoziale Krisenintervention, Interdisziplinarität, Videogestützte Psychotherapie, COVID-19 pandemic, Psychosocial care, Psychological first aid, Interdisciplinarity, Video-based psychotherapy

## Abstract

**Hintergrund:**

Die COVID-19-Pandemie hat seit ihrem Beginn zu einem erhöhten psychosozialen Unterstützungsbedarf bei Patient*innen, Angehörigen und Mitarbeiter*innen geführt und übliche Wege klinischer Versorgung erschwert. Sowohl Quarantäne- und Isolationsmaßnahmen als auch SARS-CoV-2-Infektionen und -Erkrankungen sind zu neuen und erheblichen Belastungsfaktoren geworden, die in neuen Ansätzen der Versorgung adressiert werden müssen.

**Ziel der Arbeit und Methode:**

Dieser Beitrag beschreibt die Entwicklung des Konzeptes *Psychosoziale Versorgung COVID-19* am LMU-Klinikum in München durch ein interdisziplinäres Team von Psychiater*innen, Psycholog*innen, Seelsorger*innen, Psychoonkolog*innen und Palliativmediziner*innen.

**Ergebnis:**

Das neue Versorgungsmodell zur psychosozialen Unterstützung wurde für stationäre COVID-19-Patient*innen des Klinikums, deren Angehörige und Mitarbeiter*innen bestehend aus fünf Elementen implementiert.

**Diskussion:**

Das Angebot integriert innovative und nachhaltige Ansätze, wie den Einsatz telemedizinischer Interventionen, und unterstreicht den Wert interdisziplinärer Zusammenarbeit zur Bewältigung von Herausforderungen im Gesundheitswesen.

## Abstract (en)

**Background:**

Since the beginning of the outbreak, the COVID-19 pandemic has caused an increased demand for psychosocial support for patients, their family members, and healthcare workers. Concurrently, possibilities to provide this support have been hindered. Quarantine, social isolation, and SARS-CoV‑2 infections represent new and severe stressors that have to be addressed with innovative psychosocial care.

**Objective and method:**

This article describes the COVID-19 psychosocial first aid concept at the University Hospital Munich (LMU Klinikum) developed by an interdisciplinary team of psychiatric, psychological, spiritual care, psycho-oncological, and palliative care specialists.

**Results:**

A new psychosocial first aid model has been implemented for COVID-19 inpatients, family members, and hospital staff consisting of five elements.

**Conclusion:**

The concept integrates innovative and sustainable ideas, e.g. telemedicine-based approaches and highlights the importance of multidisciplinary collaboration to cope with challenges in the healthcare system.

## Hintergrund

Die Coronavirus-disease-2019(COVID-19)-Pandemie hat sich innerhalb kurzer Zeit zu einer weltweit das Leben bestimmenden Herausforderung entwickelt, die mit negativen psychosozialen Folgen einhergeht, von denen wir alle, aber insbesondere COVID-19-erkrankte Patient*innen, deren Angehörige sowie medizinisches Personal betroffen sind [1, 2]. Dabei kann eine Vielzahl körperlicher wie psychischer Symptome auftreten; letztere reichen von diffusen Sorgen, konkreten Ängsten und depressiven Symptomen bis zu Stress und posttraumatischen Reaktionen [1, 3–5]. Bei stationär behandelten COVID-19-Patient*innen kann es neben krankheitsbezogenen Ängsten zusätzlich zu einer Belastung durch die soziale Isolation auf Spezialstationen kommen. Fehlende Besuchsmöglichkeiten für Angehörige und meist kurz gehaltene Kontakte mit medizinischem Personal in Schutzkleidung resultieren in Gefühlen von fehlender Zugehörigkeit, Überforderung, Einsamkeit und Langeweile. Zusätzlich können schwere, teils lebensbedrohliche Krankheitsverläufe über Wochen bestehen und zur Entwicklung reaktiver Belastungsstörungen sowie mittelfristig behandlungsbedürftiger neurologisch-psychiatrischer Störungsbilder führen [5]. Bei komplizierten, intensivpflichtigen Verläufen sind zudem delirante und agitierte Zustandsbilder beschrieben [6], die häufig eine Mitbetreuung durch psychiatrische Konsiliar- und Liaisondienste notwendig machen.

Für Angehörige wiederum stellen die Sorgen um den*die Erkrankte*n, die Besuchseinschränkungen und die reduzierte Austauschmöglichkeit mit dem*der Patient*in eine besondere Belastung dar. Oft besteht für sie die Notwendigkeit, sich selbst in häusliche Quarantäne und damit soziale Isolation zu begeben. Im Falle von COVID-19-bedingten Todesfällen erschweren infektiologisch notwendige Maßnahmen den Trauerprozess.

Bei medizinischem Personal können schließlich Ängste vorliegen, selbst zu erkranken bzw. die eigene Familie zu infizieren [2]. Neben der erhöhten beruflichen Belastung durch neue organisatorische Strukturen und fachspezifische Anforderungen kann es gleichzeitig zu einem Rückgang an Unterstützung und Ressourcen kommen, beispielsweise aufgrund von sozialer Ausgrenzung und Stigmatisierung, was ein wesentlicher Faktor in der Entstehung von Burn-out im Gesundheitswesen ist [7].

Negative psychosoziale Folgen wurden im Rahmen früherer Pandemien in anderen Ländern beschrieben [8] und die Notwendigkeit psychosozialer Unterstützung betont. Zugleich erschweren Pandemien aufgrund von Infektionsgefahr, Quarantäne- und Isolationsmaßnahmen deren Verfügbarkeit.

## Bausteine der Psychosozialen Versorgung COVID-19

**Figure 1:**
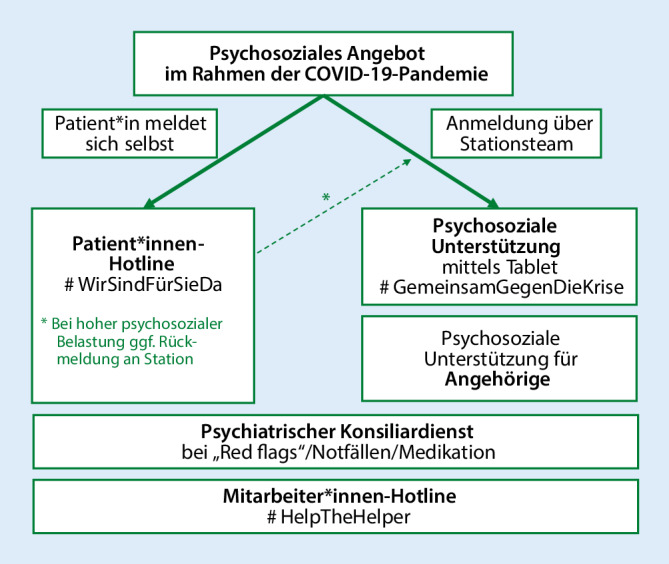

Das Konzept *Psychosoziale Versorgung COVID-19* am LMU-Klinikum wurde von einem interdisziplinären Team von Psychiater*innen, Psycholog*innen, Seelsorger*innen, Psychoonkolog*innen und Palliativmediziner*innen mit *fünf Bausteinen* entwickelt (Abb. 1):

1. Patientenhotline #WirSindFürSieDa,
2. psychosoziale Unterstützung stationärer COVID-19-Patient*innen mittels Tablet #GemeinsamGegenDieKrise,
3. psychiatrischer Konsiliardienst,
4. psychosoziale Unterstützung für Angehörige,
5. Mitarbeiter*innen-Hotline #HelpTheHelper.

Zusätzlich wurde ein Screening auf psychosoziale Belastungen (u. a. mittels Depression, Anxiety and Stress Scale [DASS-21], Impact of Event Scale-Revised [IES-R]) im Rahmen der ambulanten Nachsorge implementiert.

### Patient*innen-Hotline #WirSindFürSieDa

Die Patient*innen-Hotline wurde niederschwellig für alle Patient*innen des LMU-Klinikums eingerichtet, da auch Nicht-COVID-19-Patient*innen durch die zeitweiligen Besuchseinschränkungen erheblich belastet waren. Die Betreuung der Hotline durch die Klinikseelsorge, die bereits über die etablierte Struktur einer 24/7-Telefonbereitschaft verfügte, ermöglichte eine schnelle und zuverlässige Umsetzung mit 24-stündiger, klinikweiter Erreichbarkeit unter einer zentralen, leicht zu merkenden Durchwahl. Die hauptamtlichen Seelsorgenden waren durch ihre Expertise zeitnah in der Lage, den individuellen Versorgungsbedarf zu erheben und wo nötig und angebracht, weitere psychosoziale Anlaufstellen einzuschalten. Auch die Möglichkeit einer längerfristigen telefonischen Begleitung durch den Stamm der erfahrenen ehrenamtlichen Seelsorgenden wurde vorbereitet.

### Psychosoziale Unterstützung von COVID-19-Patient*innen mittels Tablet #GemeinsamGegenDieKrise

Auf den COVID-19-Stationen des Klinikums wurde für stationäre Patient*innen mittels Videosprechstunde ein spezielles psychotherapeutisches Angebot mit supportiven Kurzinterventionen geschaffen. Inhaltlich wurde als Gesprächsleitfaden das BELLA-Konzept für psychologische Kriseninterventionen [9] mit einer ressourcenorientieren Grundhaltung herangezogen (Tab. 1 und Fallbeispiel Infobox 1). Neben dem Beziehungsaufbau, der mittels Videosprechstunde erfahrungsgemäß schneller gelingt als am Telefon, konnte durch Validierung von Emotionen und Ressourcenaktivierung bereits in vielen Fällen eine emotionale Entlastung der Patient*innen erreicht werden. Neben Achtsamkeits- und Imaginationsübungen zur Entspannung (Cave: atemzentrierte Übungen bei Dyspnoe) wurde der*die Patient*in zu eigenständigen Übungen wie beispielsweise Anti-Grübel-Strategien oder dem „butterfly hug“, einer aus dem EMDR stammenden Technik zur Selbstberuhigung [10], angeleitet.

**Table 1:** 

*Beziehung aufbauen*	Eigene Person und Funktion vorstellen
	Bequeme Gesprächssituation für Patient schaffen
*Erfassen der Situation*	Aktuelle Situation, vorliegende Emotionen und Bedürfnisse beschreiben lassen
	Auf Ängste, Depression, posttraumatische Belastungssymptome, Eigengefährdung screenen
	Achtsamkeitstechniken einsetzen, die Nähe trotz der Distanz schaffen: „Was sehen Sie um sich herum? Was hören Sie?“
*Linderung der Symptome*	Emotional validieren und entlasten
	Psychoedukation zu Stressreaktionen
	Individuell angepasste Interventionen wie Achtsamkeit (Cave: Atmung), Imaginationsübungen, „butterfly hug“, kognitive Umstrukturierung
*Leute und Dinge zur Unterstützung einbeziehen*	Ressourcen erfragen
	Soziales Netz aktivieren
	Evtl. Stationspersonal einbeziehen
*Abschluss*	Weiteres Vorgehen klären/Folgetermin
	Zu Angeboten nach Entlassung beraten
	Evtl. psychiatrischen Konsiliardienst einbeziehen

#### Infobox 1 Fallbeispiel tabletgestützte Patient*innen-Intervention (Dauer 40 min)

Der 46-jährige Patient wird initial vom Rettungsdienst aufgrund progredienter Dyspnoe in das LMU-Klinikum gebracht und ist bereits nach kurzer Zeit intubationspflichtig. Der COVID-19-Abstrich auf der Intensivstation ist positiv. Der weitere Verlauf gestaltet sich protrahiert und mit Komplikationen. Dennoch kann der Patient im weiteren Verlauf extubiert und schließlich auch entisoliert werden. Aufgrund von Ängsten und Sorgen des Patienten wegen des langen Aufenthalts auf der Intensivstation wird die Anfrage zur psychosozialen Mitbetreuung gestellt. Die Station ist hierfür mit einem Tablet ausgestattet, das dem Patienten ins Zimmer gereicht wird.

Der Patient schildert, dass er durch den nur langsam vorangehenden Genesungserlauf sehr belastet sei. Da er bisher nur selten und für kurze Zeit krank gewesen sei, verliere er „das Vertrauen in seinen Körper“. Auf Station komme ihm alles „fremdbestimmt“ vor. Die geschilderten Eindrücke und Beschwerden des Patienten werden als nachvollziehbar eingeordnet und validiert. Der Patient wirkt hierdurch etwas entlastet, sodass weitere psychosoziale Belastungsfaktoren erfragt werden können. Es zeigt sich, dass der Patient bereits vor dem Klinikaufenthalt mit Problemen konfrontiert war (u. a. Sorge um Arbeitsplatzverlust), um die er sich aktuell nicht kümmern kann und daher auch Hilflosigkeit verspüre. Außerdem vermisse er seine Ehefrau und die Kinder, die wegen der Besuchseinschränkung nicht auf der Station vorbeikommen können. Im weiteren Gesprächsverlauf wird auf funktionale Strategien zum Umgang mit der aktuellen Situation auf der Station eingegangen und dysfunktionalen Strategien gegenübergestellt. Der Patient wird darin bekräftigt, sich auf eine achtsame und annehmende Grundhaltung einzulassen („Ich kümmere mich um das Hier und Jetzt“, „Alles kann sich schnell ändern“). Zusätzlich werden Möglichkeiten gesammelt, die Abläufe auf der Station möglichst aktiv mitzugestalten und Ressourcen zur Unterstützung zu aktivieren (u. a. der Kontakt zur Familie und das soziale Netz). Zuletzt wird ein Folgetermin vereinbart und der Patient wirkt zu Gesprächsende sichtlich gelöst.

Vermittelt durch die COVID-19-Stationen wurden werktags Termine mit Psycholog*innen und Ärzt*innen der Klinik für Psychiatrie und Psychotherapie vergeben, die sich zeitweise selbst im Homeoffice befanden. Notfallmäßig bestand eine Rufbereitschaft über das Team der Psycho-Onkologie (in Kooperation mit lebensmut e. V.). Helfer*innen hatten im April/Mai 2020 die Möglichkeit, an einer Schulung und anschließend wöchentlicher, webbasierter Supervision teilzunehmen. Der Umgang mit schwierigen Therapiesituationen und die fachgerechte Durchführung der Interventionen wurden vermittelt. Als wertvoll erachtet wurden beispielsweise die „Do’s and Don’ts der Gesprächsgestaltung via Tablet“ [11]. Zusätzlich wurden Gespräche anonymisiert protokolliert und evaluiert, um den Bedarf anzupassen.

Zur Durchführung des Angebots erhielt die Klinik Anfang April 2020 eine Spende von 60 Tabletgeräten, die von Mitarbeiter*innen Münchner Unternehmen zur Verfügung gestellt und auf COVID-19-Stationen verteilt wurden. Die Tablets konnten auch für die Kommunikation der Patient*innen mit ihren Angehörigen eingesetzt werden und enthielten vorinstallierte Podcasts mit Entspannungsübungen und Informationsmaterial. Die von der Kassenärztlichen Bundesvereinigung zertifizierte Videosprechstunde ermöglichte eine sichere Peer-to-peer-Verbindung und die Verwendung fast aller internetfähigen Endgeräte, d. h. sowohl des*der Patient*in als auch des Behandelnden (beispielsweise im Homeoffice), was die Erreichbarkeit und Durchführbarkeit des Angebots weiter erleichterte.

### Psychiatrischer Konsiliardienst

Beim Vorliegen von „red flags“ (z. B. Suizidalität, Delir, Notwendigkeit psychopharmakologischer Behandlung) konnte der psychiatrische Konsiliardienst in die Behandlung von COVID-19-Patient*innen einbezogen werden. Bei den strengen Infektionsschutzmaßnahmen erleichterten die Tablets auch hier die interdisziplinäre Kommunikation zwischen Psychiater*innen, Patient*innen und den COVID-19-Behandlungsteams.

### Psychosoziale Unterstützung für Angehörige

Das Beratungsangebot für Angehörige von COVID-19-Patient*innen beinhaltete lösungsorientierte, supportive Gespräche. Auch eine Weitervermittlung an interne und externe Hilfsangebote (z. B. Sozialdienst, Seelsorge, Familiensprechstunde, niedergelassene Psychotherapeut*innen) fand nach Bedarf statt. Das Angebot für Angehörige baute auf die bestehenden Strukturen der Psycho-Onkologie auf und wurde wochentags vom psychologischen Team der Klinik für Anästhesiologie, Klinik und Poliklinik für Orthopädie, Physikalische Medizin und Rehabilitation sowie der Psycho-Onkologie in Kooperation mit lebensmut e. V. gewährleistet. Außerhalb dieser Zeiten bestand eine notfallmäßige Rufbereitschaft, sodass Angehörige 24/7 versorgt werden konnten.

### Psychosoziale Hotline für Mitarbeiter*innen #HelpTheHelper

Ergänzend zu bereits bestehenden Maßnahmen der Gesundheitsförderung (Teambesprechungen, Mitarbeiter*innen-Gespräche, Angebote zur Kinderbetreuung usw.) wurde mit der #HelpTheHelper-Hotline ein niederschwelliges und anonymes Angebot eingerichtet. Neben der Entlastung der medizinischen Teams vor Ort (u. a. 1700 Mediziner*innen und 3200 Pflegekräfte) war das Angebot explizit auch an nichtmedizinische Mitarbeiter*innen (z. B. Reinigungspersonal, technische Mitarbeiter*innen, Verwaltungsfachkräfte) sowie Mitarbeiter*innen im Krankenstand oder Homeoffice gerichtet. Ein nach dem BELLA-Konzept (Tab. 1) geschultes Team psychologischer und ärztlicher Mitarbeiter*innen war täglich von 8 bis 20 Uhr auf einer zentralen Nummer auch von außerhalb des Klinikums erreichbar (Fallbeispiel Infobox 2). Durch die Einrichtung der Mobiltelefone über die Medizin- und Informationstechnik des LMU-Klinikums war dem Hotlineteam hierbei eine flexible Übernahme von Telefondiensten aus dem Homeoffice möglich. In wöchentlichen videobasierten Supervisionen wurden die geführten Telefonate strukturiert anhand eines anonymisierten Dokumentationsbogens nachbesprochen und der Versorgungsbedarf evaluiert. Neben der direkt-supportiven Funktion diente die Hotline der Identifikation von Mitarbeiter*innen mit interventionsbedürftigen psychischen Beschwerden. In Zusammenarbeit mit der Ambulanz der Klinik für Psychiatrie und Psychotherapie fand die Vermittlung einer regelmäßigen (evtl. videobasierten) psychotherapeutischen und/oder psychiatrischen Versorgung statt.

#### Infobox 2 Fallbeispiel Mitarbeiter*innen-Hotline (Dauer 30 min)

Die 50-jährige Anruferin stellt sich als Krankenschwester vor. Sie sei bereits seit annähernd 20 Jahren am Klinikum beschäftigt, aktuell jedoch aufgrund einer chronischen Erkrankung und dem damit verbundenen Gesundheitsrisiko im Falle einer COVID-19-Infektion krankgeschrieben. Eine Kollegin habe ihr vorgeschlagen, bei der psychosozialen Hotline anzurufen. Sie selbst wolle auf keinen Fall wertvolle Zeit in Anspruch nehmen: „Bestimmt gibt es gerade viele Anrufer mit viel schlimmeren Sorgen?“ Nach Zuspruch des Hotlinemitarbeiters schildert die Anruferin folgende Beschwerden: Seit zwei Woche leide sie unter wiederkehrenden Beklemmungsgefühlen in der Brust, Luftnot und Schweißausbrüchen. Zudem leide sie unter ständigen Ängsten, sich anstecken zu können. Sie habe das Gefühl, von Bekannten gemieden zu werden, da sie in einer Klinik arbeite. Die Versorgung mit dem „Nötigsten“ erledige sie daher selbst, verlasse ihre Wohnung ansonsten kaum. Zunehmend habe sie das Gefühl, sich nicht mehr entspannen zu können. Sie sei es nicht gewohnt, so viel Zeit in der eigenen Wohnung zu verbringen. Die regelmäßigen Abläufe und Aufgaben des Klinikalltags würden fehlen. „Ich habe 20 Jahre Berufserfahrung, meine Kolleginnen können mich jetzt bestimmt gut gebrauchen!“ Der Hotlinemitarbeiter validiert die Beschwerden als situationsbedingt nachvollziehbar und nimmt eine emotionale Entlastung im Verlauf des Gesprächs wahr. Es stellt sich heraus, dass die Anruferin sich auf entsprechenden Informationsseiten des Klinikums sowie dem Internet bereits gut über Hinweise zum Umgang mit Stress informiert hat. Dennoch können gemeinsam einige individuelle Bewältigungsstrategien identifiziert werden: regelmäßiges Spazierengehen zu wenig frequentierten Tageszeiten, telefonischer Austausch mit den Stationskolleginnen, aktives Ansprechen von Sorgen gegenüber Bekannten, Vereinbarung eines Mitarbeitergesprächs mit der Vorgesetzten, Nutzung zur Verfügung gestellter Entspannungsübungen. Abschließend wird die Anruferin über die Möglichkeit eines erneuten Anrufes sowie über weitere psychosoziale Unterstützungsmöglichkeiten auch außerhalb des Klinikums informiert.

### Nachsorge

Aufgrund der möglichen zeitlichen Verzögerung psychosozialer Belastungssymptome wurden im Rahmen der ambulanten Nachsorge von COVID-19-Patient*innen Screeninginstrumente implementiert, die u. a. ängstliche und depressive Symptome (DASS-21) sowie posttraumatische Belastungssymptomatik (IES-R) erfassen. Belastete Patient*innen können so weiterhin frühzeitig identifiziert werden und individuell psychosoziale Unterstützung beispielsweise durch die Klinik für Psychiatrie und Psychotherapie erhalten. Der Umfang dieses Nachsorgebedarfs wird sich abhängig vom Verlauf der COVID-19-Pandemie in Deutschland erst zeigen.

## Implementierung, Nutzung und Bedarfssteuerung

Die Entwicklung und Implementierung des Angebots erfolgte durch ein interdisziplinäres Team und wurde in wöchentlichen Sitzungen koordiniert (Tab. 2 für eine Schätzung der Implementierungskosten). Als Investitionskosten fielen die Beschaffung von Tablets und Smartphones an, auf denen die kostenlose Videosprechstunde installiert wurde. Zusätzlich wurde über das Klinische Arbeitsplatzsystem die Möglichkeit eingerichtet, klinikweit Aufträge an das psychosoziale Modul zu stellen. Das Angebot wurde mittels verschiedener Werbemaßnahmen bekannt gemacht (Flyer, Intranet, Informationsvideos, E‑Mail, persönliche Vorstellung und technische Unterstützung auf den COVID-19-Stationen). Das inhaltliche Konzept wurde in zwei webbasierten Schulungen vermittelt (à 90 min) und eine wöchentliche Supervisionsmöglichkeit angeboten (à 60 min). Personell wurde das Angebot durch vorhandenes Personal und ehrenamtliche Helfer*innen bereitgestellt, welches aufgrund der Corona-bedingten, reduzierten Belegung der Kliniken bzw. aus dem Homeoffice über zeitliche Kapazitäten verfügte. Es zeigte sich eine gute Akzeptanz des Angebots bei COVID-19-Patient*innen und Mitarbeiter*innen mit einem typischen zeitlichen Umfang pro Anfrage von ca. 30 min (exklusive Vor- und Nachbereitung). So war das Angebot vor allem für respiratorisch stabile Patient*innen nutzbar.

**Table 2:** 

	Anteil Arbeitszeit	Monatliches Bruttogehalt (€)	Projektbezogene Kosten (€)	Gesamtkosten (€)
*Baustein 1: Patientenhotline*				
Team der Seelsorge	0,2	^a^	^a^	^a^
*Bausteine 2 und 5: Tabletgestützte Videotherapie und Mitarbeiterhotline*				*7620*
Oberarzt	0,2	8925	1785	–
Leitende Psychologin	0,2	5999	1200	–
Arzt in Weiterbildung	0,5	5210	2605	–
Psychologin	0,5	4060	2030	–
*Baustein 3: Psychiatrischer Konsiliardienst*				*1339*
Oberärztin	0,15	8925	1339	–
*Baustein 4: Angehörigenunterstützung*^b^				*2597*
Oberärztin	0,2	8925	1785	–
Psychologin	0,2	4060	812	–
*Investitionskosten*				*3000*
10 Tablets^c^	–	–	2000	–
5 Smartphones	–	–	1000	–
*Summe:*				*14.556*

^a^Diese Stellen werden am LMU-Klinikum über die Evangelisch-Lutherische Kirche in Bayern und die Erzdiözese München Freising finanziert

^b^Finanziert über Spenden von lebensmut e. V.

^c^Finanziert durch Firmenspenden, evtl. höherer Bedarf an Geräten

**Figure 2:**
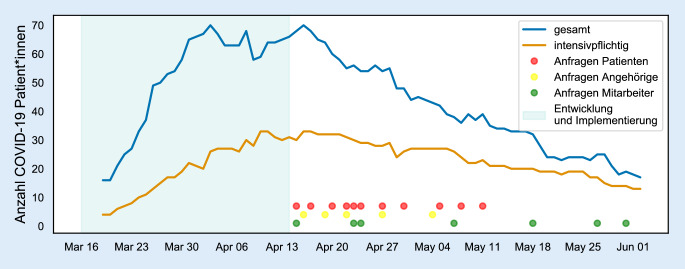

Die im internationalen Vergleich geringen Infektionszahlen in Deutschland im Frühjahr 2020 spiegelten sich auch in der Belegung mit COVID-19-Patient*innen innerhalb des Klinikums wider, welche ab Mai 2020 stetig abnahmen (Abb. 2). Hierauf wurde mit einer zeitlichen Reduktion des Angebots reagiert (beispielsweise reduzierte Erreichbarkeit der Mitarbeiter*innen-Hotline). Eine erneute Erweiterung des Angebots im Falle erneut steigender Infektionszahlen ist aufgrund der nun fest implementierten Strukturen sowie der auftragsbasierten Koordination der Bausteine flexibel möglich.

## Diskussion

Zusammenfassend hat sich gezeigt, dass zu Beginn der COVID-19-Pandemie innerhalb kürzester Zeit ein effizientes neues Modul zur psychosozialen Versorgung von COVID-19-Patient*innen, Angehörigen und Klinikpersonal aufgebaut werden konnte. Hierbei hat sich insbesondere die interdisziplinäre Zusammenarbeit als hilfreich erwiesen, um verfügbare personelle und materielle Klinikressourcen schnell zu mobilisieren und bestehende Expertise im Umgang mit Krisensituationen zu bündeln. Zusätzlich signalisierte in kurzer Zeit eine Vielzahl an Helfer*innen die Bereitschaft, in der psychosozialen Versorgung (z. T. auch aus dem Homeoffice heraus) mitzuwirken. Sie wurden speziell in Techniken der psychologischen Gesprächsführung und Krisenintervention sowie im Einsatz digitaler Interventionen geschult, was zu einem Gewinn an Fertigkeiten führte.

Die Nutzung und Anpassung etablierter Strukturen (z. B. Anfrage des Angebots über das Klinische Arbeitsplatzsystem) erleichterte die Kommunikation mit den Stationen.

Eine vielversprechende, auch langfristig nutzbare Neuerung bietet der Einsatz von Tablets im Klinikalltag, welche im Rahmen des Moduls erstmalig klinikübergreifend eingesetzt wurden. Im Falle eines besonderen Infektionsschutzes bot die Videosprechstunde eine sichere neue Behandlungsoption für Patient*innen (z. B. auf Transplantationsstationen) oder Klinikpersonal (z. B. schwangere Mitarbeiterinnen).

Da aufgrund der Infektionsschutzgebote auch reguläre Einzel- und Gruppentherapie aktuell oft nur mit Mund-Nasen-Schutz (MNS) durchführbar sind und dieser mit eingeschränkter Wahrnehmbarkeit der Mimik erfahrungsgemäß die Kommunikation zwischen Patient und Therapeut verändert, bietet Videotelefonie evtl. sogar eine interessante Alternative zum regulären Psychotherapiesetting mit MNS. Diese Unterschiede der Patienten-Therapeuten-Kommunikation sind jedoch bislang kaum wissenschaftlich untersucht.

Durch die COVID-19-Pandemie ist in kurzer Zeit eine Vielzahl hilfreicher Arbeitsmaterialien entstanden, die zumeist kostenfrei verfügbar sind (siehe beispielhaft [12–14]). Wie auch unser Versorgungskonzept ermöglichen diese im Falle eines erneuten Anstiegs der Infektionszahlen eine zeitnahe Reaktion auf psychosoziale Belastungen.

Einschränkend ist zu sagen, dass das beschriebene Projekt auf die Strukturen eines multidisziplinären Universitätsklinikums zurückgreift und somit möglicherweise nicht auf jeden Versorgungskontext übertragbar ist. Durch die transparente Angabe der Entwicklungs- und Implementierungskosten unseres Angebots bieten wir hier eine Orientierungsmöglichkeit für andere Einrichtungen der Primärversorgung. Zudem war eine kontrollierte Testung der Wirksamkeit der beschriebenen Angebote aufgrund der akuten Entwicklung der COVID-19-Pandemie nicht möglich.

Das beschriebene Modul stellt ein praxisnahes Beispiel für die zeitnahe, pragmatische Etablierung eines nachhaltigen Versorgungsangebots dar, das durch interdisziplinäre Zusammenarbeit sowie die Integration bestehender und telemedizinischer Angebote verschiedene Aspekte psychosozialer Belastung adäquat bedienen und auf den sich verändernden Bedarf flexibel reagieren kann.

## Fazit für die Praxis

- Anhaltende Krisensituationen, wie die aktuelle Corona-Pandemie, gehen mit erhöhten psychosozialen Belastungen für Patient*innen, deren Angehörige sowie Mitarbeiter*innen im Gesundheitswesen einher.
- Interdisziplinäre Zusammenarbeit ermöglicht an Kliniken der Primärversorgung die zeitnahe Umsetzung psychosozialer Unterstützungsangebote.
- Die Integration neuer telemedizinischer Versorgungsansätze (v. a. tabletbasierte Videosprechstunde) in bestehende Strukturen erscheint praktikabel und sinnvoll.
- Hierbei ist auch auf die Translation neu geschaffener Strukturen über die Krisensituation hinweg zu achten.
